# 2-[4-(2-Meth­oxy­phen­yl)piperazin-1-yl]-*N*-(pyridin-2-yl)acetamide

**DOI:** 10.1107/S1600536810053067

**Published:** 2010-12-24

**Authors:** Chunxiong Lu, Quanfu Jiang

**Affiliations:** a Key Laboratory of Nuclear Medicine, Ministry of Health, Jiangsu Key Laboratory of Molecular Nuclear Medicine, Jiangsu Institute of Nuclear Medicine, Wuxi 214063, People’s Republic of China

## Abstract

In the title compound, C_18_H_22_N_4_O_2_, the piperizine ring adopts a chair conformation and the dihedral angle between the pyridine and benzene rings is 67.6 (9)°. The conformations of the attachment of the anisole and *N*-ethyl­pyridin-2-amine groups to the piperazine ring are (+)anti­periplanar. Intra­molecular C—H⋯O and N—H⋯N inter­actions occur. In the crystal, inter­molecular C—H⋯N hydrogen bonds are present. There are two crystallographically independent but identical mol­ecules per asymmetric unit.

## Related literature

For the use of the title compound in the synthesis of receptor imaging agents, see: Lebars *et al.* (1998 ▶); Zhuang *et al.* (1994 ▶).
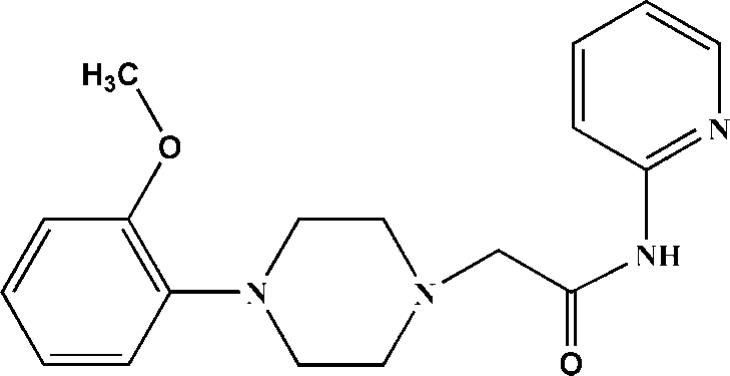


## Experimental

### 

#### Crystal data

C_18_H_22_N_4_O_2_
*M* *_r_* = 326.40
Triclinic, 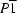

*a* = 11.595 (4) Å
*b* = 12.382 (4) Å
*c* = 14.073 (4) Å
α = 106.228 (4)°
β = 91.767 (3)°
γ = 114.627 (2)°
*V* = 1738.2 (9) Å^3^
*Z* = 4
Mo *K*α radiation
μ = 0.08 mm^−1^
*T* = 143 K
0.21 × 0.17 × 0.09 mm

#### Data collection

Rigaku AFC10/Saturn724+ diffractometer
16957 measured reflections
7823 independent reflections
4689 reflections with *I* > 2σ(*I*)
*R*_int_ = 0.041

#### Refinement

*R*[*F* ^2^ > 2σ(*F* ^2^)] = 0.055
*wR*(*F* ^2^) = 0.134
*S* = 1.00
7823 reflections
443 parameters
H atoms treated by a mixture of independent and constrained refinement
Δρ_max_ = 0.74 e Å^−3^
Δρ_min_ = −0.19 e Å^−3^

### 

Data collection: *CrystalClear* (Rigaku, 2008 ▶); cell refinement: *CrystalClear*; data reduction: *CrystalClear*; program(s) used to solve structure: *SHELXS97* (Sheldrick, 2008 ▶); program(s) used to refine structure: *SHELXL97* (Sheldrick, 2008 ▶); molecular graphics: *SHELXTL* (Sheldrick, 2008 ▶); software used to prepare material for publication: *SHELXL97*.

## Supplementary Material

Crystal structure: contains datablocks I, global. DOI: 10.1107/S1600536810053067/fk2033sup1.cif

Structure factors: contains datablocks I. DOI: 10.1107/S1600536810053067/fk2033Isup2.hkl

Additional supplementary materials:  crystallographic information; 3D view; checkCIF report

## Figures and Tables

**Table 1:** Hydrogen-bond geometry (Å, °)

*D*—H⋯*A*	*D*—H	H⋯*A*	*D*⋯*A*	*D*—H⋯*A*
N3—H03⋯N2	0.85 (2)	2.20 (3)	2.692 (3)	117.0 (19)
N7—H07⋯N6	0.86 (2)	2.26 (3)	2.734 (3)	115.3 (19)
C2—H2⋯N8	0.95	2.44	3.354 (3)	161
C14—H14⋯O2	0.95	2.33	2.923 (3)	120
C20—H20⋯N4	0.95	2.59	3.527 (3)	168

**Fig. 1.**
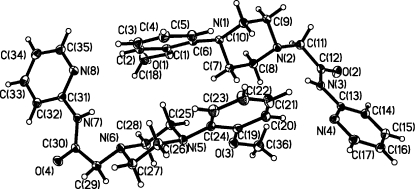
A view of the title compound with the atomic numbering scheme with displacement ellipsoids drawn at the 50% probability level.

## supplementary crystallographic information

### Comment

2-(4-(2-methoxyphenyl)piperazin-1-yl)-*N*-(pyridin-2-yl)acetamide, (I), is an important intermediate product in the synthesis of 131I-MPPI (Zhuang *et al.*, 1994) and 18 F-MPPF (Lebars *et al.*, 1998), serotonin(5-HT1A) receptor imaging agents (131I-MPPI = 4-(2'-methoxypheny)-1-[2'-(N-2''-pyridinyl)- *p*-131I-iodobenzamido]ethyl-piperazine and 18 F-MPPF = 4-(2'-methoxyphenyl)-1-[2'-(N-2''-pyridinyl) -*p*-18 F-fluorobenzamido]ethylpiperazine). We report here the crystal structure of (I).hydrate (Fig. 1). The molecule of (I) consists of an anisole and an *N*-ethylpyridin-2-amine arms connected to a piperazine ring. The piperazine ring adopts a chair conformation. The dihedral angle between the phenyl and pyridine rings is 67.6 (9)°. The conformations of the attachment of the anisole and *N*-ethylpyridin-2-amine groups to the piperazine ring are best described by the torsion angles of 171.99 (18)° and -174.56 (18)° for C6—N1—C7—C8 and C11—N2—C9—C10, respectively; *i.e.* they adopt +antiperiplanar conformations. The molecules show intra- and intermolecular hydrogen-bonding interactions of types N—H···N, C—H···N and C—H···O (Table 1).

### Experimental

The title compound was synthesized according to the method reported in the literature (Zhuang *et al.*, 1994) and crystallized from a mixed solvent composed of acetone and water (1:1); colourless block-shaped crystals were obtained after several days.

### Refinement

The amino H atoms were located in a difference Fourier map and refined with N—H distance restrained to 0.85 Å. Positional parameters of all the H atoms bonded to C atoms were calculated geometrically and were allowed to ride on the C atoms to which they were bonded, with C—H distances of 0.95Å (CH), 0.98Å (CH_3_) or 0.99Å (CH_2_), and with *U*_iso_(H) =1.2 or 1.5 (methyl) *U*_eq_ of the parent atoms.

### Crystal data

**Table:**

C_18_H_22_N_4_O_2_	*Z* = 4
*M**_r_* = 326.40	*F*(000) = 696
Triclinic, *P*1	*D*_x_ = 1.247 Mg m^−^^3^
Hall symbol: -P 1	Mo *K*α radiation, λ = 0.71073 Å
*a* = 11.595 (4) Å	Cell parameters from 3991 reflections
*b* = 12.382 (4) Å	θ = 3.0–27.5°
*c* = 14.073 (4) Å	µ = 0.08 mm^−^^1^
α = 106.228 (4)°	*T* = 143 K
β = 91.767 (3)°	Prism, colourless
γ = 114.627 (2)°	0.21 × 0.17 × 0.09 mm
*V* = 1738.2 (9) Å^3^	

### Data collection

**Table:**

Rigaku AFC10/Saturn724+ diffractometer	4689 reflections with *I* > 2σ(*I*)
Radiation source: Rotating Anode	*R*_int_ = 0.041
graphite	θ_max_ = 27.5°, θ_min_ = 3.0°
Detector resolution: 28.5714 pixels mm^-1^	*h* = −15→15
φ and ω scans	*k* = −16→12
16957 measured reflections	*l* = −18→18
7823 independent reflections	

### Refinement

**Table:**

Refinement on *F*^2^	Primary atom site location: structure-invariant direct methods
Least-squares matrix: full	Secondary atom site location: difference Fourier map
*R*[*F*^2^ > 2σ(*F*^2^)] = 0.055	Hydrogen site location: difmap and geom
*wR*(*F*^2^) = 0.134	H atoms treated by a mixture of independent and constrained refinement
*S* = 1.00	*w* = 1/[σ^2^(*F*_o_^2^) + (0.0569*P*)^2^ + 0.168*P*] where *P* = (*F*_o_^2^ + 2*F*_c_^2^)/3
7823 reflections	(Δ/σ)_max_ = 0.001
443 parameters	Δρ_max_ = 0.74 e Å^−^^3^
0 restraints	Δρ_min_ = −0.19 e Å^−^^3^

### Special details

**Table:**

Geometry. All e.s.d.'s (except the e.s.d. in the dihedral angle between two l.s. planes) are estimated using the full covariance matrix. The cell e.s.d.'s are taken into account individually in the estimation of e.s.d.'s in distances, angles and torsion angles; correlations between e.s.d.'s in cell parameters are only used when they are defined by crystal symmetry. An approximate (isotropic) treatment of cell e.s.d.'s is used for estimating e.s.d.'s involving l.s. planes.
Refinement. Refinement of *F*^2^ against ALL reflections. The weighted *R*-factor *wR* and goodness of fit *S* are based on *F*^2^, conventional *R*-factors *R* are based on *F*, with *F* set to zero for negative *F*^2^. The threshold expression of *F*^2^ > σ(*F*^2^) is used only for calculating *R*-factors(gt) *etc*. and is not relevant to the choice of reflections for refinement. *R*-factors based on *F*^2^ are statistically about twice as large as those based on *F*, and *R*- factors based on ALL data will be even larger.

### Fractional atomic coordinates and isotropic or equivalent isotropic displacement parameters (Å^2^)

**Table:**

	*x*	*y*	*z*	*U*_iso_*/*U*_eq_	
O1	−0.16031 (14)	0.19320 (13)	0.60110 (10)	0.0318 (4)	
O2	0.26509 (14)	0.03711 (15)	0.17233 (10)	0.0389 (4)	
N1	0.04151 (16)	0.31688 (15)	0.51774 (11)	0.0248 (4)	
N2	0.15938 (16)	0.22961 (16)	0.35729 (11)	0.0264 (4)	
N3	0.35747 (16)	0.17050 (17)	0.33185 (12)	0.0260 (4)	
N4	0.54677 (16)	0.24391 (16)	0.43628 (12)	0.0290 (4)	
C1	−0.0797 (2)	0.31511 (19)	0.65726 (14)	0.0263 (4)	
C2	−0.0993 (2)	0.3747 (2)	0.75015 (14)	0.0304 (5)	
H2	−0.1719	0.3312	0.7774	0.037*	
C3	−0.0128 (2)	0.4982 (2)	0.80355 (16)	0.0371 (5)	
H3	−0.0263	0.5386	0.8673	0.044*	
C4	0.0919 (2)	0.5616 (2)	0.76425 (16)	0.0388 (6)	
H4	0.1507	0.6459	0.8005	0.047*	
C5	0.1115 (2)	0.5019 (2)	0.67106 (15)	0.0330 (5)	
H5	0.1846	0.5461	0.6446	0.040*	
C6	0.02656 (19)	0.37892 (19)	0.61564 (13)	0.0254 (4)	
C7	0.0808 (2)	0.2177 (2)	0.51565 (14)	0.0291 (5)	
H7A	0.0225	0.1601	0.5484	0.035*	
H7B	0.1693	0.2557	0.5533	0.035*	
C8	0.0760 (2)	0.14470 (19)	0.40775 (14)	0.0297 (5)	
H8A	0.1047	0.0796	0.4070	0.036*	
H8B	−0.0136	0.1023	0.3713	0.036*	
C9	0.1161 (2)	0.3261 (2)	0.35931 (14)	0.0310 (5)	
H9A	0.0260	0.2858	0.3243	0.037*	
H9B	0.1705	0.3827	0.3240	0.037*	
C10	0.1254 (2)	0.4008 (2)	0.46679 (14)	0.0304 (5)	
H10A	0.2155	0.4413	0.5018	0.036*	
H10B	0.0987	0.4675	0.4684	0.036*	
C11	0.1642 (2)	0.1621 (2)	0.25557 (13)	0.0297 (5)	
H11A	0.1808	0.2184	0.2144	0.036*	
H11B	0.0788	0.0898	0.2263	0.036*	
C12	0.26561 (19)	0.11417 (19)	0.24878 (14)	0.0270 (5)	
C13	0.47825 (19)	0.17094 (19)	0.34499 (14)	0.0244 (4)	
C14	0.5227 (2)	0.1054 (2)	0.27087 (15)	0.0326 (5)	
H14	0.4697	0.0520	0.2078	0.039*	
C15	0.6471 (2)	0.1202 (2)	0.29197 (16)	0.0367 (5)	
H15	0.6816	0.0783	0.2425	0.044*	
C16	0.7206 (2)	0.1964 (2)	0.38561 (16)	0.0346 (5)	
H16	0.8062	0.2084	0.4015	0.042*	
C17	0.6659 (2)	0.2542 (2)	0.45484 (16)	0.0336 (5)	
H17	0.7153	0.3045	0.5197	0.040*	
C18	−0.2720 (2)	0.1297 (2)	0.64012 (17)	0.0411 (6)	
H18A	−0.2460	0.1235	0.7045	0.062*	
H18B	−0.3236	0.0453	0.5926	0.062*	
H18C	−0.3234	0.1767	0.6500	0.062*	
H03	0.345 (2)	0.224 (2)	0.3780 (15)	0.036 (7)*	
O3	0.31024 (14)	0.19997 (13)	0.69524 (10)	0.0328 (4)	
O4	−0.20714 (14)	0.09195 (14)	1.08091 (10)	0.0364 (4)	
N5	0.27157 (15)	0.34038 (15)	0.86559 (11)	0.0247 (4)	
N6	0.07490 (15)	0.27036 (16)	0.98664 (11)	0.0258 (4)	
N7	−0.17632 (17)	0.22583 (16)	0.99004 (12)	0.0261 (4)	
N8	−0.31000 (17)	0.26615 (17)	0.89803 (12)	0.0318 (4)	
C19	0.37328 (19)	0.3275 (2)	0.71544 (14)	0.0278 (5)	
C20	0.4550 (2)	0.3846 (2)	0.65566 (15)	0.0341 (5)	
H20	0.4689	0.3348	0.5965	0.041*	
C21	0.5165 (2)	0.5145 (2)	0.68257 (17)	0.0442 (6)	
H21	0.5720	0.5534	0.6414	0.053*	
C22	0.4978 (2)	0.5871 (2)	0.76827 (18)	0.0459 (6)	
H22	0.5411	0.6760	0.7869	0.055*	
C23	0.4155 (2)	0.5301 (2)	0.82765 (16)	0.0362 (5)	
H23	0.4025	0.5812	0.8865	0.043*	
C24	0.35148 (19)	0.4006 (2)	0.80341 (14)	0.0266 (5)	
C25	0.13434 (19)	0.26517 (19)	0.82010 (14)	0.0276 (5)	
H25A	0.1261	0.2088	0.7520	0.033*	
H25B	0.0960	0.3215	0.8139	0.033*	
C26	0.0632 (2)	0.1882 (2)	0.88466 (14)	0.0301 (5)	
H26A	−0.0288	0.1382	0.8539	0.036*	
H26B	0.0991	0.1293	0.8883	0.036*	
C27	0.21204 (19)	0.3438 (2)	1.03070 (14)	0.0302 (5)	
H27A	0.2496	0.2865	1.0353	0.036*	
H27B	0.2215	0.3994	1.0994	0.036*	
C28	0.2834 (2)	0.4222 (2)	0.96688 (14)	0.0311 (5)	
H28A	0.2471	0.4808	0.9634	0.037*	
H28B	0.3753	0.4723	0.9977	0.037*	
C29	0.0039 (2)	0.1977 (2)	1.04953 (14)	0.0296 (5)	
H29A	0.0444	0.2460	1.1206	0.036*	
H29B	0.0133	0.1189	1.0326	0.036*	
C30	−0.1381 (2)	0.16446 (19)	1.04003 (14)	0.0264 (5)	
C31	−0.30054 (19)	0.21492 (19)	0.96784 (13)	0.0250 (4)	
C32	−0.4019 (2)	0.15893 (19)	1.01496 (15)	0.0300 (5)	
H32	−0.3907	0.1248	1.0652	0.036*	
C33	−0.5194 (2)	0.1543 (2)	0.98670 (16)	0.0346 (5)	
H33	−0.5907	0.1163	1.0174	0.042*	
C34	−0.5328 (2)	0.2049 (2)	0.91386 (16)	0.0362 (5)	
H34	−0.6130	0.2019	0.8929	0.043*	
C35	−0.4262 (2)	0.2599 (2)	0.87240 (16)	0.0369 (5)	
H35	−0.4350	0.2956	0.8227	0.044*	
C36	0.3320 (2)	0.1232 (2)	0.60821 (16)	0.0408 (6)	
H36A	0.3084	0.1408	0.5487	0.061*	
H36B	0.2793	0.0346	0.6007	0.061*	
H36C	0.4231	0.1414	0.6154	0.061*	
H07	−0.118 (2)	0.271 (2)	0.9629 (15)	0.032 (6)*	

### Atomic displacement parameters (Å^2^)

**Table:**

	*U*^11^	*U*^22^	*U*^33^	*U*^12^	*U*^13^	*U*^23^
O1	0.0284 (8)	0.0330 (8)	0.0296 (7)	0.0089 (7)	0.0087 (6)	0.0107 (6)
O2	0.0337 (9)	0.0433 (10)	0.0289 (8)	0.0161 (8)	0.0021 (6)	−0.0025 (7)
N1	0.0269 (9)	0.0278 (9)	0.0236 (8)	0.0136 (8)	0.0063 (7)	0.0114 (7)
N2	0.0258 (9)	0.0322 (10)	0.0233 (8)	0.0134 (8)	0.0046 (7)	0.0109 (7)
N3	0.0252 (10)	0.0298 (10)	0.0227 (9)	0.0137 (8)	0.0045 (7)	0.0054 (8)
N4	0.0278 (10)	0.0306 (10)	0.0279 (9)	0.0126 (8)	0.0011 (7)	0.0095 (7)
C1	0.0273 (11)	0.0306 (11)	0.0258 (10)	0.0162 (9)	0.0028 (8)	0.0110 (9)
C2	0.0292 (12)	0.0393 (13)	0.0298 (11)	0.0185 (10)	0.0089 (9)	0.0154 (10)
C3	0.0426 (14)	0.0390 (14)	0.0304 (11)	0.0226 (12)	0.0086 (10)	0.0049 (10)
C4	0.0404 (14)	0.0320 (13)	0.0383 (12)	0.0153 (11)	0.0069 (10)	0.0043 (10)
C5	0.0297 (12)	0.0317 (12)	0.0349 (11)	0.0107 (10)	0.0081 (9)	0.0112 (10)
C6	0.0261 (11)	0.0331 (12)	0.0231 (10)	0.0164 (10)	0.0049 (8)	0.0124 (9)
C7	0.0319 (12)	0.0320 (12)	0.0301 (11)	0.0170 (10)	0.0083 (9)	0.0150 (9)
C8	0.0297 (12)	0.0288 (12)	0.0326 (11)	0.0142 (10)	0.0073 (9)	0.0106 (9)
C9	0.0313 (12)	0.0395 (13)	0.0329 (11)	0.0193 (11)	0.0105 (9)	0.0209 (10)
C10	0.0325 (12)	0.0290 (12)	0.0327 (11)	0.0143 (10)	0.0105 (9)	0.0129 (9)
C11	0.0234 (11)	0.0397 (13)	0.0245 (10)	0.0133 (10)	0.0015 (8)	0.0099 (9)
C12	0.0247 (11)	0.0288 (11)	0.0235 (10)	0.0075 (9)	0.0052 (8)	0.0093 (9)
C13	0.0235 (11)	0.0261 (11)	0.0254 (10)	0.0093 (9)	0.0068 (8)	0.0131 (8)
C14	0.0313 (12)	0.0410 (13)	0.0283 (11)	0.0180 (11)	0.0073 (9)	0.0117 (10)
C15	0.0353 (13)	0.0464 (15)	0.0371 (12)	0.0232 (12)	0.0140 (10)	0.0171 (11)
C16	0.0243 (12)	0.0376 (13)	0.0461 (13)	0.0123 (10)	0.0074 (9)	0.0214 (11)
C17	0.0289 (12)	0.0350 (12)	0.0346 (11)	0.0116 (10)	−0.0006 (9)	0.0127 (10)
C18	0.0376 (14)	0.0358 (14)	0.0423 (13)	0.0059 (11)	0.0162 (10)	0.0166 (11)
O3	0.0421 (10)	0.0291 (8)	0.0297 (8)	0.0180 (7)	0.0115 (6)	0.0088 (6)
O4	0.0331 (9)	0.0422 (9)	0.0377 (8)	0.0140 (8)	0.0107 (7)	0.0224 (7)
N5	0.0219 (9)	0.0281 (9)	0.0215 (8)	0.0093 (8)	0.0031 (6)	0.0070 (7)
N6	0.0203 (9)	0.0317 (10)	0.0236 (8)	0.0092 (8)	0.0041 (6)	0.0096 (7)
N7	0.0221 (9)	0.0293 (10)	0.0272 (9)	0.0091 (8)	0.0068 (7)	0.0129 (8)
N8	0.0339 (11)	0.0384 (11)	0.0279 (9)	0.0182 (9)	0.0064 (7)	0.0142 (8)
C19	0.0257 (11)	0.0303 (12)	0.0288 (10)	0.0139 (10)	0.0020 (8)	0.0095 (9)
C20	0.0315 (13)	0.0424 (14)	0.0299 (11)	0.0162 (11)	0.0085 (9)	0.0137 (10)
C21	0.0377 (15)	0.0455 (15)	0.0464 (14)	0.0100 (12)	0.0181 (11)	0.0225 (12)
C22	0.0468 (16)	0.0318 (13)	0.0498 (14)	0.0065 (12)	0.0155 (12)	0.0160 (11)
C23	0.0349 (13)	0.0293 (12)	0.0390 (12)	0.0093 (10)	0.0092 (10)	0.0105 (10)
C24	0.0210 (11)	0.0314 (12)	0.0272 (10)	0.0107 (9)	0.0042 (8)	0.0105 (9)
C25	0.0241 (11)	0.0309 (12)	0.0243 (10)	0.0096 (9)	0.0012 (8)	0.0082 (9)
C26	0.0259 (11)	0.0326 (12)	0.0266 (10)	0.0091 (10)	0.0025 (8)	0.0082 (9)
C27	0.0231 (11)	0.0389 (13)	0.0250 (10)	0.0128 (10)	0.0013 (8)	0.0070 (9)
C28	0.0237 (11)	0.0328 (12)	0.0284 (10)	0.0081 (10)	0.0036 (8)	0.0049 (9)
C29	0.0286 (12)	0.0370 (13)	0.0270 (10)	0.0166 (10)	0.0047 (8)	0.0125 (9)
C30	0.0297 (12)	0.0265 (11)	0.0215 (9)	0.0112 (10)	0.0046 (8)	0.0075 (8)
C31	0.0238 (11)	0.0255 (11)	0.0235 (9)	0.0112 (9)	0.0025 (8)	0.0043 (8)
C32	0.0270 (12)	0.0296 (12)	0.0321 (11)	0.0109 (10)	0.0077 (9)	0.0105 (9)
C33	0.0270 (12)	0.0298 (12)	0.0415 (12)	0.0103 (10)	0.0088 (9)	0.0065 (10)
C34	0.0287 (13)	0.0388 (14)	0.0390 (12)	0.0178 (11)	0.0009 (9)	0.0053 (10)
C35	0.0367 (14)	0.0467 (15)	0.0329 (11)	0.0246 (12)	0.0040 (9)	0.0117 (10)
C36	0.0558 (17)	0.0380 (14)	0.0356 (12)	0.0285 (13)	0.0134 (11)	0.0094 (11)

### Geometric parameters (Å, °)

**Table:**

O1—C1	1.374 (2)	O3—C19	1.372 (2)
O1—C18	1.426 (2)	O3—C36	1.427 (2)
O2—C12	1.220 (2)	O4—C30	1.223 (2)
N1—C6	1.433 (2)	N5—C24	1.421 (2)
N1—C10	1.469 (2)	N5—C28	1.464 (2)
N1—C7	1.470 (3)	N5—C25	1.476 (2)
N2—C11	1.458 (2)	N6—C29	1.464 (2)
N2—C8	1.469 (2)	N6—C27	1.469 (2)
N2—C9	1.470 (3)	N6—C26	1.473 (2)
N3—C12	1.355 (2)	N7—C30	1.352 (3)
N3—C13	1.404 (3)	N7—C31	1.406 (3)
N3—H03	0.85 (2)	N7—H07	0.86 (2)
N4—C13	1.337 (2)	N8—C31	1.334 (3)
N4—C17	1.343 (3)	N8—C35	1.348 (3)
C1—C2	1.387 (3)	C19—C20	1.389 (3)
C1—C6	1.406 (3)	C19—C24	1.414 (3)
C2—C3	1.394 (3)	C20—C21	1.388 (3)
C2—H2	0.9500	C20—H20	0.9500
C3—C4	1.374 (3)	C21—C22	1.372 (3)
C3—H3	0.9500	C21—H21	0.9500
C4—C5	1.391 (3)	C22—C23	1.387 (3)
C4—H4	0.9500	C22—H22	0.9500
C5—C6	1.391 (3)	C23—C24	1.388 (3)
C5—H5	0.9500	C23—H23	0.9500
C7—C8	1.521 (3)	C25—C26	1.513 (3)
C7—H7A	0.9900	C25—H25A	0.9900
C7—H7B	0.9900	C25—H25B	0.9900
C8—H8A	0.9900	C26—H26A	0.9900
C8—H8B	0.9900	C26—H26B	0.9900
C9—C10	1.510 (3)	C27—C28	1.515 (3)
C9—H9A	0.9900	C27—H27A	0.9900
C9—H9B	0.9900	C27—H27B	0.9900
C10—H10A	0.9900	C28—H28A	0.9900
C10—H10B	0.9900	C28—H28B	0.9900
C11—C12	1.517 (3)	C29—C30	1.513 (3)
C11—H11A	0.9900	C29—H29A	0.9900
C11—H11B	0.9900	C29—H29B	0.9900
C13—C14	1.386 (3)	C31—C32	1.388 (3)
C14—C15	1.387 (3)	C32—C33	1.381 (3)
C14—H14	0.9500	C32—H32	0.9500
C15—C16	1.386 (3)	C33—C34	1.378 (3)
C15—H15	0.9500	C33—H33	0.9500
C16—C17	1.375 (3)	C34—C35	1.377 (3)
C16—H16	0.9500	C34—H34	0.9500
C17—H17	0.9500	C35—H35	0.9500
C18—H18A	0.9800	C36—H36A	0.9800
C18—H18B	0.9800	C36—H36B	0.9800
C18—H18C	0.9800	C36—H36C	0.9800
			
C1—O1—C18	116.84 (15)	C19—O3—C36	117.38 (16)
C6—N1—C10	114.88 (16)	C24—N5—C28	115.19 (16)
C6—N1—C7	113.42 (15)	C24—N5—C25	114.62 (15)
C10—N1—C7	110.02 (15)	C28—N5—C25	109.58 (14)
C11—N2—C8	111.99 (16)	C29—N6—C27	110.89 (15)
C11—N2—C9	112.14 (16)	C29—N6—C26	111.33 (16)
C8—N2—C9	109.09 (15)	C27—N6—C26	108.63 (15)
C12—N3—C13	129.39 (18)	C30—N7—C31	128.76 (18)
C12—N3—H03	113.4 (15)	C30—N7—H07	114.5 (15)
C13—N3—H03	116.0 (15)	C31—N7—H07	116.3 (15)
C13—N4—C17	116.92 (17)	C31—N8—C35	116.63 (18)
O1—C1—C2	123.16 (18)	O3—C19—C20	123.64 (18)
O1—C1—C6	116.37 (16)	O3—C19—C24	115.77 (17)
C2—C1—C6	120.46 (19)	C20—C19—C24	120.6 (2)
C1—C2—C3	120.11 (19)	C21—C20—C19	119.8 (2)
C1—C2—H2	119.9	C21—C20—H20	120.1
C3—C2—H2	119.9	C19—C20—H20	120.1
C4—C3—C2	120.17 (19)	C22—C21—C20	120.5 (2)
C4—C3—H3	119.9	C22—C21—H21	119.8
C2—C3—H3	119.9	C20—C21—H21	119.8
C3—C4—C5	119.7 (2)	C21—C22—C23	119.7 (2)
C3—C4—H4	120.2	C21—C22—H22	120.1
C5—C4—H4	120.2	C23—C22—H22	120.1
C4—C5—C6	121.55 (19)	C22—C23—C24	121.8 (2)
C4—C5—H5	119.2	C22—C23—H23	119.1
C6—C5—H5	119.2	C24—C23—H23	119.1
C5—C6—C1	118.02 (17)	C23—C24—C19	117.59 (18)
C5—C6—N1	122.69 (17)	C23—C24—N5	122.76 (18)
C1—C6—N1	119.26 (18)	C19—C24—N5	119.57 (19)
N1—C7—C8	110.03 (16)	N5—C25—C26	109.92 (16)
N1—C7—H7A	109.7	N5—C25—H25A	109.7
C8—C7—H7A	109.7	C26—C25—H25A	109.7
N1—C7—H7B	109.7	N5—C25—H25B	109.7
C8—C7—H7B	109.7	C26—C25—H25B	109.7
H7A—C7—H7B	108.2	H25A—C25—H25B	108.2
N2—C8—C7	110.27 (16)	N6—C26—C25	110.55 (17)
N2—C8—H8A	109.6	N6—C26—H26A	109.5
C7—C8—H8A	109.6	C25—C26—H26A	109.5
N2—C8—H8B	109.6	N6—C26—H26B	109.5
C7—C8—H8B	109.6	C25—C26—H26B	109.5
H8A—C8—H8B	108.1	H26A—C26—H26B	108.1
N2—C9—C10	109.63 (16)	N6—C27—C28	110.30 (16)
N2—C9—H9A	109.7	N6—C27—H27A	109.6
C10—C9—H9A	109.7	C28—C27—H27A	109.6
N2—C9—H9B	109.7	N6—C27—H27B	109.6
C10—C9—H9B	109.7	C28—C27—H27B	109.6
H9A—C9—H9B	108.2	H27A—C27—H27B	108.1
N1—C10—C9	109.40 (16)	N5—C28—C27	109.87 (17)
N1—C10—H10A	109.8	N5—C28—H28A	109.7
C9—C10—H10A	109.8	C27—C28—H28A	109.7
N1—C10—H10B	109.8	N5—C28—H28B	109.7
C9—C10—H10B	109.8	C27—C28—H28B	109.7
H10A—C10—H10B	108.2	H28A—C28—H28B	108.2
N2—C11—C12	114.41 (16)	N6—C29—C30	115.62 (17)
N2—C11—H11A	108.7	N6—C29—H29A	108.4
C12—C11—H11A	108.7	C30—C29—H29A	108.4
N2—C11—H11B	108.7	N6—C29—H29B	108.4
C12—C11—H11B	108.7	C30—C29—H29B	108.4
H11A—C11—H11B	107.6	H29A—C29—H29B	107.4
O2—C12—N3	125.0 (2)	O4—C30—N7	125.2 (2)
O2—C12—C11	121.35 (18)	O4—C30—C29	119.63 (19)
N3—C12—C11	113.57 (17)	N7—C30—C29	115.06 (17)
N4—C13—C14	123.7 (2)	N8—C31—C32	123.6 (2)
N4—C13—N3	112.45 (17)	N8—C31—N7	112.81 (17)
C14—C13—N3	123.79 (18)	C32—C31—N7	123.57 (19)
C13—C14—C15	117.8 (2)	C33—C32—C31	118.1 (2)
C13—C14—H14	121.1	C33—C32—H32	121.0
C15—C14—H14	121.1	C31—C32—H32	121.0
C16—C15—C14	119.6 (2)	C34—C33—C32	119.7 (2)
C16—C15—H15	120.2	C34—C33—H33	120.1
C14—C15—H15	120.2	C32—C33—H33	120.1
C17—C16—C15	117.9 (2)	C35—C34—C33	117.9 (2)
C17—C16—H16	121.0	C35—C34—H34	121.0
C15—C16—H16	121.0	C33—C34—H34	121.0
N4—C17—C16	124.0 (2)	N8—C35—C34	124.0 (2)
N4—C17—H17	118.0	N8—C35—H35	118.0
C16—C17—H17	118.0	C34—C35—H35	118.0
O1—C18—H18A	109.5	O3—C36—H36A	109.5
O1—C18—H18B	109.5	O3—C36—H36B	109.5
H18A—C18—H18B	109.5	H36A—C36—H36B	109.5
O1—C18—H18C	109.5	O3—C36—H36C	109.5
H18A—C18—H18C	109.5	H36A—C36—H36C	109.5
H18B—C18—H18C	109.5	H36B—C36—H36C	109.5
			
C18—O1—C1—C2	3.2 (3)	C36—O3—C19—C20	0.6 (3)
C18—O1—C1—C6	−176.80 (19)	C36—O3—C19—C24	−178.73 (19)
O1—C1—C2—C3	179.3 (2)	O3—C19—C20—C21	−179.0 (2)
C6—C1—C2—C3	−0.7 (3)	C24—C19—C20—C21	0.3 (3)
C1—C2—C3—C4	0.3 (3)	C19—C20—C21—C22	0.5 (4)
C2—C3—C4—C5	−0.2 (4)	C20—C21—C22—C23	−0.9 (4)
C3—C4—C5—C6	0.6 (4)	C21—C22—C23—C24	0.6 (4)
C4—C5—C6—C1	−1.0 (3)	C22—C23—C24—C19	0.2 (3)
C4—C5—C6—N1	177.4 (2)	C22—C23—C24—N5	176.9 (2)
O1—C1—C6—C5	−178.98 (18)	O3—C19—C24—C23	178.73 (19)
C2—C1—C6—C5	1.0 (3)	C20—C19—C24—C23	−0.6 (3)
O1—C1—C6—N1	2.6 (3)	O3—C19—C24—N5	1.9 (3)
C2—C1—C6—N1	−177.39 (18)	C20—C19—C24—N5	−177.44 (19)
C10—N1—C6—C5	−18.5 (3)	C28—N5—C24—C23	−13.1 (3)
C7—N1—C6—C5	109.2 (2)	C25—N5—C24—C23	115.4 (2)
C10—N1—C6—C1	159.80 (18)	C28—N5—C24—C19	163.57 (18)
C7—N1—C6—C1	−72.5 (2)	C25—N5—C24—C19	−67.9 (2)
C6—N1—C7—C8	171.99 (16)	C24—N5—C25—C26	170.29 (17)
C10—N1—C7—C8	−57.8 (2)	C28—N5—C25—C26	−58.4 (2)
C11—N2—C8—C7	176.23 (16)	C29—N6—C26—C25	178.58 (17)
C9—N2—C8—C7	−59.0 (2)	C27—N6—C26—C25	−59.0 (2)
N1—C7—C8—N2	57.8 (2)	N5—C25—C26—N6	59.0 (2)
C11—N2—C9—C10	−174.60 (16)	C29—N6—C27—C28	−177.92 (16)
C8—N2—C9—C10	60.7 (2)	C26—N6—C27—C28	59.4 (2)
C6—N1—C10—C9	−171.08 (16)	C24—N5—C28—C27	−170.09 (17)
C7—N1—C10—C9	59.5 (2)	C25—N5—C28—C27	58.9 (2)
N2—C9—C10—N1	−61.1 (2)	N6—C27—C28—N5	−60.2 (2)
C8—N2—C11—C12	−86.9 (2)	C27—N6—C29—C30	154.65 (17)
C9—N2—C11—C12	150.02 (18)	C26—N6—C29—C30	−84.3 (2)
C13—N3—C12—O2	11.1 (3)	C31—N7—C30—O4	−4.8 (3)
C13—N3—C12—C11	−164.95 (19)	C31—N7—C30—C29	179.19 (18)
N2—C11—C12—O2	166.17 (18)	N6—C29—C30—O4	171.57 (17)
N2—C11—C12—N3	−17.6 (2)	N6—C29—C30—N7	−12.2 (2)
C17—N4—C13—C14	1.1 (3)	C35—N8—C31—C32	−1.1 (3)
C17—N4—C13—N3	−177.56 (17)	C35—N8—C31—N7	−179.91 (18)
C12—N3—C13—N4	175.9 (2)	C30—N7—C31—N8	−165.16 (19)
C12—N3—C13—C14	−2.7 (3)	C30—N7—C31—C32	16.0 (3)
N4—C13—C14—C15	−2.3 (3)	N8—C31—C32—C33	1.1 (3)
N3—C13—C14—C15	176.1 (2)	N7—C31—C32—C33	179.82 (18)
C13—C14—C15—C16	1.5 (3)	C31—C32—C33—C34	−0.2 (3)
C14—C15—C16—C17	0.5 (3)	C32—C33—C34—C35	−0.6 (3)
C13—N4—C17—C16	1.1 (3)	C31—N8—C35—C34	0.2 (3)
C15—C16—C17—N4	−1.9 (3)	C33—C34—C35—N8	0.7 (3)

### Hydrogen-bond geometry (Å, °)

**Table:**

*D*—H···*A*	*D*—H	H···*A*	*D*···*A*	*D*—H···*A*
N3—H03···N2	0.85 (2)	2.20 (3)	2.692 (3)	117.0 (19)
N7—H07···N6	0.86 (2)	2.26 (3)	2.734 (3)	115.3 (19)
C2—H2···N8	0.95	2.44	3.354 (3)	161
C7—H7A···O1	0.99	2.42	3.010 (3)	118
C14—H14···O2	0.95	2.33	2.923 (3)	120
C20—H20···N4	0.95	2.59	3.527 (3)	168
C25—H25A···O3	0.99	2.34	2.950 (3)	119
C32—H32···O4	0.95	2.34	2.917 (3)	118
